# Gastrointestinal parasites of captive and free-ranging wild animals in the state of Mato Grosso do Sul

**DOI:** 10.1590/S1984-29612025030

**Published:** 2025-06-13

**Authors:** Angela Maria da Silva, Jordana Toqueto, Nayara Carvalho, Dirceu Guilherme de Souza Ramos, Andréia Lima Tomé Melo

**Affiliations:** 1 Programa de Pós-graduação em Biociência Animal, Universidade de Cuiabá – UNIC, Cuiabá, MT, Brasil; 2 Laboratório de Análises Clínica Veterinárias Hemathus, Dourados, MT, Brasil; 3 Centro de Reabilitação de Animais Silvestres – CRAS do Estado do Mato Grosso do Sul, Campo Grande, MT, Brasil; 4 Instituto do Meio Ambiente de Dourados – IMAM, Dourados, MT, Brasil; 5 Programa de Pós-graduação em Biociência Animal, Instituto de Ciências Agrárias, Universidade Federal de Jataí – UFJ, Jataí, GO, Brasil; 6 Programa de Pós-graduação em Ciências Aplicadas à Saúde, Instituto de Ciências da Saúde, Universidade Federal de Jataí – UFJ, Jataí, GO, Brasil; 7 Laboratório de Parasitologia e Análises Clínicas Veterinária, Instituto de Ciências Agrárias, Universidade Federal de Jataí – UFJ, Jataí, GO, Brasil

**Keywords:** Wild animals, conservation, Helminths, Protozoa, Animais selvagens, conservação, Helmintofauna, Protozoários

## Abstract

Wild animals are hosts for many species of parasites which act as opportunistic or primary agents of disease. This study investigated the presence of endoparasites in the gastrointestinal tracts of wild animals from the State of Mato Grosso do Sul in the Cerrado, Atlantic Forest, and Pantanal biomes. From October 2022 to June 2024, fecal samples were collected from wild animals at two locations: Wildlife Rehabilitation Center (CRAS) in Campo Grande and the Paragem Municipal Natural Park (PNMP) in Dourados. A total of 109 fecal samples were collected: 96 from CRAS and 13 from PNMP and techniques of flotation and sedimentation were used. The prevalence of parasitism was 51.04% and 23.07% in the captive and free-living animals, respectively. The parasites found were nematodes (Strongyloidea, Ancylostomatidae, Ascaridida, Ascaropsinae, *Toxocara* spp., Capillarinae, *Trichuris* spp., *Ascaris suum*, Oxyurida, and nematode larvae), cestodes (*Dipylidium* spp. and *Spirometra* spp.), trematodes (*Paragonimus* spp.), and protozoa (*Entamoeba* spp., *Eimeria* spp., *Cystoisospora* spp., and Coccidia). The highest prevalence was observed for helminths of the Strongyloidea and Ancylostomatidae, followed by Coccidia and *Entamoeba* spp.. We highlight the importance of such studies to better understand the circulation of etiological agents that may pose a risk to animal and human health.

## Resumo

Animais selvagens são hospedeiros de muitas espécies de parasitas que atuam como agentes oportunistas ou primários de doenças. Este estudo investigou a presença de endoparasitas no trato gastrointestinal de animais selvagens do Estado do Mato Grosso do Sul, nos biomas Cerrado, Mata Atlântica e Pantanal. De outubro de 2022 a junho de 2024, amostras fecais foram coletadas de animais selvagens em dois locais: Centro de Reabilitação de Animais Silvestres (CRAS), em Campo Grande, e Parque Natural Municipal da Paragem (PNMP), em Dourados. Ao todo, 109 amostras fecais foram coletadas: 96 do CRAS e 13 do PNMP, utilizando-se as técnicas de flotação e sedimentação. A prevalência de parasitismo foi de 51,04% e 23,07% nos animais cativos e de vida livre, respectivamente. Os parasitas encontrados foram nematoides (Strongyloidea, Ancylostomatidae, Ascaridida, Ascaropsinae, *Toxocara* spp., Capillarinae, *Trichuris* spp., *Ascaris suum*, Oxyurida e larvas); cestódeos (*Dipylidium* spp. e *Spirometra* spp.), trematódeos (*Paragonimus* spp.) e protozoários (*Entamoeba* spp., *Eimeria* spp., *Cystoisospora* spp. e Coccidia). A maior prevalência foi observada para helmintos de Strongyloidea e Ancylostomatidae, seguidos por Coccidia e *Entamoeba* spp.. Destaca-se a importância desses estudos para melhor compreender a circulação de agentes etiológicos que podem representar risco à saúde animal e humana.

## Introduction

Wild animals are hosts for a wide variety of parasites that can act as opportunistic or primary agents of disease (Freitas et al., 2001, 2002; Godoy & Cubas, 2011). More than 120 parasitic diseases caused by arthropods, insects, helminths, and/or protozoa have been reported in wild, domestic, and human animals; some of these parasites are known for their impact on human health and the health of parasitized animals (CDC, 2024a). Despite considerable technological and scientific advances in veterinary medicine, parasitic diseases are still considered a serious health problem for animals and humans, especially zoonotic parasitic species (Joy et al., 2008).

Parasitism results from the presence of macro-and microparasites and constitutes one of the most serious problems in conservation veterinary medicine (Zapalski & Hubert, 2011). It also threatens carnivore populations, because parasites can actively act in the structuring of animal communities, affecting the relative abundance of different species in the same way that a predator can affect them. This justifies the inclusion of parasitism as a biotic force capable of determining the biodiversity of communities (Poulin, 1999).

In contrast, parasites can play a beneficial role in the ecosystem, generating a balance between host and parasite, and can be an indicator of the health of a location. The use of soil fauna as a bioindicator of soil quality is recent (Pulleman et al., 2012) and the environmental impact and evidence of environmental health through species that affect habitat changes by altering their numbers, physiology or chemical composition are being extensively studied (Sures, 2003). For example, helminths, which are parasites of terrestrial and aquatic organisms, are considered useful bioindicators because they are affected by both natural and anthropogenic environments (Vidal-Martínez et al., 2006).

Identifying feces in the environment of some rare animal species makes it difficult to observe them, and this, together with other factors such as the behavior of burying feces or defecating in water or on tree branches, represents a great challenge (Chame, 2003; Chame & Sianto, 2014) Even so, it is worth noting that feces are the most evident and easily recognizable sign (Liebenberg, 2014). Therefore, to obtain more information about the biological and pathological processes of parasitism, it is necessary to know, correctly identify and categorize the species of parasites (Vieira, 2011).

To obtain more information about the biological and pathological processes of parasitism, it is necessary to know, correctly identify, and categorize parasite species (Vieira, 2011). Given the importance of understanding the parasites that affect wild animals and the scarcity of studies on this subject, this study investigated the presence of endoparasites in the gastrointestinal tracts of captive and free-living animals in the state of Mato Grosso do Sul in the Cerrado, Atlantic Forest, and Pantanal biomes, thus generating relevant information about the parasitic ecology of the specimens sampled in this study.

## Material and Methods

### Study locations

Fecal samples were collected from two locations in Mato Grosso do Sul. Those from captive wild animals were collected at the Wildlife Rehabilitation Center (CRAS) (20°27’05”S and 54°33’54”W), located in Campo Grande, coming from 18 municipalities, while those from free-living animals were obtained from the Paragem Municipal Natural Park (22°15’21”S and 54°47’39”W) in the municipality of Dourados. The animals sampled at CRAS were rescued from various locations in the state, such as Dourados, Rio Verde, Campo Grande, Bataguassu, Rio Brilhante, Nova Andradina, Sidrolândia, Inocência, Aquidauana, São Gabriel do Oeste, Corguinho, Anastácio, Caarapó, Fátima do Sul, Jateí, Nova Alvorada do Sul, Bonito, and Corumbá.

### Parasitological sampling and diagnosis

Samples were collected from the enclosure in which the animals were kept, in the morning before cleaning, and without handling the animals. The feces were visibly fresh, with a shiny and moist appearance. Priority was given to collection from the top of the fecal mass to avoid contact and contamination with the enclosure of the animals in captivity. For birds, samples were collected on the paper lining the floor of the cages.

For samples collected from free-living animals, the same collection criteria were adopted, focusing on fresh feces, with a moist and shiny appearance, and without contact with the animals. Samples collected from the environment were evaluated for their appearance and morphology to identify the host species, in addition to an analysis carried out by park biologists who identified the samples based on their knowledge of the species present in the park.

The collected samples were stored in collection tubes, identified, and kept in a refrigerated environment at an average temperature of 4 °C. Each sample was identified with a date and location record and sent in isothermal boxes on ice to the laboratory for analysis. Some captive animal enclosures contained more than one animal and a pool of fecal samples was collected.

From October 2022 to June 2024, 109 fecal samples were collected: 96 samples from animals from CRAS, Campo Grande, and 13 samples from free-living animals in the Paragem Municipal Natural Park, Dourados. The fecal samples were sent to the Veterinary Clinical Analysis Laboratory (HEMATHUS) in Dourados, where they were subjected to coproparasitological examination.

The coproparasitological techniques used were the Willis flotation (Willis, 1921) and the spontaneous sedimentation of Hoffman et al. (1934). Subsequently, a frosted microscope slide was positioned in direct contact with the liquid and the sample examined under a Nikon E200 optical microscope at 100x and 400x magnification. Morphological identification of parasites was performed as described by Foreyt (2001) and Zajac & Conboy (2011).

### Data analysis

The data were processed using Microsoft Excel® spreadsheets and organized by collection number and identification, egg types, and life stage (larvae and adults) of the parasites. In addition, relative frequency calculations were performed for each parasitic form. To infer the levels of parasite infection, comparisons of the relative frequencies identified with those of other studies on both free-living and captive species were used.

### Ethical and legal aspects

This study was granted environmental authorization for scientific research in Conservation Units by the Mato Grosso do Sul Environmental Institute, Imasul (process number 71041982/2022). As no vertebrate animals were manipulated, approval from the Animal Ethics Committee was not required.

## Results

Coproparasitological examinations indicated that the occurrence of parasitism in the captive animals was 51.04% (49/96), whereas that in the free-living animals was 23.07% (3/13). The identified gastrointestinal nematodes were Ancylostomatidae, Ascaridida, Ascaropsinae, Capillarinae, Strongyloidea, *Ascaris suum*, *Trichuris* spp., *Toxocara* spp., and Oxyurida spp. The trematodes found belonged to the genus *Paragonimus* and other genera that could not be identified by coproparasitological examination alone. Cestodes, such as *Dipylidium* spp., *Spirometra* spp., and others, whose genera could not be identified using these coproparasitological examination methods, were identified.

**Figure 1:**
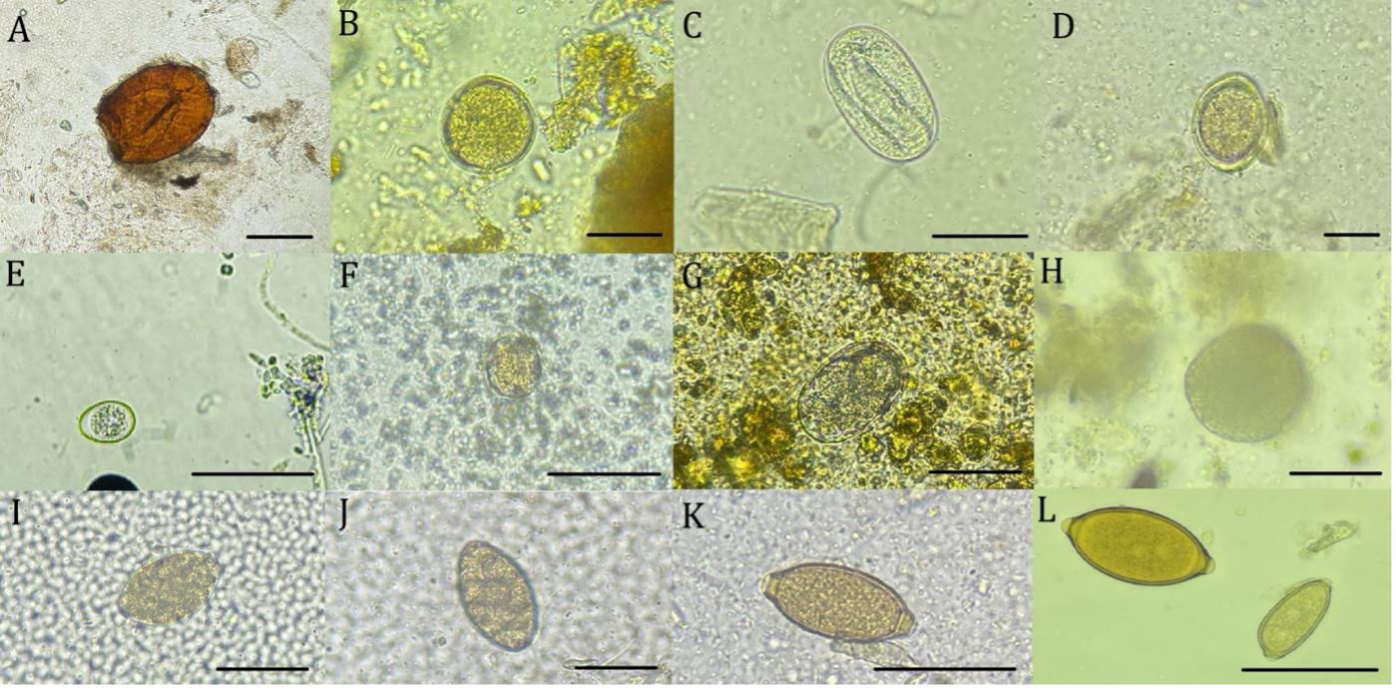
Eggs and oocysts of gastrointestinal parasites in fecal samples from wild animals in the state of Mato Grosso do Sul (scale bar reference). (A) *Paragonimus* spp. egg (50µm), (B) *Toxocara* spp. egg (50µm), (C) Strongyloidea larvae egg (50µm), (D) Ascaropsinae egg (50µm), (E) *Eimeria* spp. oocyst (50µm), (F) Cestoda egg (50µm), (G) Ancylostomatidae egg (50µm), (H) *Ascaris suum* egg (50µm), (I) Cestoda egg (50µm), (J) *Spirometra* spp. egg (50µm), (K) *Trichuris* spp. egg (50µm), (L) Eggs of *Trichuris* spp. and Capillarinae (50µm).

The protozoa found included *Entamoeba* spp., *Cystoisospora* spp., *Eimeria* spp., and other Coccidia species (Tables 1 and 2). Five enclosures had more than one animal species, making it necessary to create a sample pool of the collected feces. It was not possible to identify which animal the excreta belonged to, as they did not defecate at the time of collection. The animals in the enclosures shared between different species were not present, and at the time of collection, pathological changes compatible with parasitic diseases were found in some analyses. Figure 1 shows the helminth eggs and protozoan oocysts identified in this study.

**Table 1:** Feces of captive animals collected at CRAS in Campo Grande, Mato Grosso do Sul, and results of coproparasitological analyses carried out between 2022 and 2024.

**Hosts**	**Parasites identified**	**Positive/tested**
**Class Mammalia**		
**ORDER ARTIODACTYLA**		
*Dicotyles tajacu*	Strongyloidea^a,b^	3/5
	*Ascaris suum* ^a^	2/5
	Ascaropsinae^a^	1/5
	Coccidia^b^	1/5
*Mazama gouazoubira*	Cestoda	1/3
**ORDER CARNIVORA**		
*Cerdocyon thous*	*Toxocara* spp.	1/4
	*Cystoisospora* spp.	2/4
	Capillarinae^c^	1/4
	Strongyloidea^c^	1/4
	*Trichuris* spp.^c^	1/4
*Chrysocyon brachyurus*	Ancylostomatidae^d^	1/1
	*Toxocara* spp.^d^	1/1
*Herpailurus yagouaroundi*	Ancylostomatidae^e^	1/2
	*Dipylidium* spp.^e^	1/2
	*Toxocara* spp.^e^	1/2
	*Cystoisospora* spp.^f^	1/2
	Strongyloidea^f^	1/2
*Leopardus pardalis*	Ancylostomatidae	1/1
*Leopardus tigrinus*	-	-/2
*Lycalopex vetulus*	Ancylostomatidae	1/1
*Nasua nasua*	-	-/2
*Procyon cancrivorus*	Ascaridida^g^	1/1
	Cestoda^g^	1/1
	Coccidia^g^	1/1
	*Spirometra* spp.^g^	1/1
	Strongyloidea^g^	1/1
	*Trichuris* spp.^g^	1/1
*Puma concolor*	*Toxocara* spp.	1/3
**ORDER DIDELPHIMORPHIA**		
*Didelphis albiventris*	Strongyloidea	1/3
**ORDER PERISSODACTYLA**		
*Tapirus terrestris*	*Entamoeba* spp.	1/3
	Ascaropsinae^h^	1/3
	Strongyloidea^h^	1/3
**ORDER PILOSA**		
*Myrmecophaga tridactyla*	Nematoda	1/1
**ORDER PRIMATA**		
*Alouatta caraya*	Ancylostomatidae^i^	1/4
	Oxyurida^i^	1/4
	*Paragonimus* spp. ^i^	2/4
*Callithrix penicillata*	-	-/1
*Sapajus* spp.	Oxyurida^j^	1/5
	Strongyloidea^j^	2/5
	Coccidia	1/5
**ORDER RODENTIA**		
*Dasyprocta azarae*	Trematoda	1/2
*Hydrochoerus hydrochaeris*	Strongyloidea	1/2
	Coccidia^k^	1/2
	Nematoda^k^	1/2
**SAMPLE POOL ENCLOSURE A**		
*Leopardus pardalis*	Ancylostomatidae	1/1
*Puma concolor*	*Toxocara* spp.	1/1
**SAMPLE POOL ENCLOSURE B**		
*Cerdocyon thous*	Ancylostomatidae	1/1
*Lycalopex vetulus*	*Toxocara* spp.	1/1
		
**Class Aves**		
**ORDER COLUMBIFORMES**		
*Zenaida auriculata*	-	-/2
**ORDER FALCONIFORMES**		
*Caracara plancus*	-	-/1
**ORDER GALLIFORMES**		
*Crax fasciolata*	-	-/1
**ORDER PASSERIFORMES**		
*Cyanocompsa brissonii*	*Entamoeba* spp.	1/2
*Oryzoborus angolensis*	*Entamoeba* spp.	1/6
	*Eimeria* spp.	1/6
	Coccidia	1/6
*Oryzoborus maximiliani*	*Entamoeba* spp.	4/9
	Cestoda	1/9
*Paroaria coronata*	-	-/1
*Paroaria dominicana*	-	-/1
*Serinus canaria domestica*	*Entamoeba* spp.	1/1
*Sicalis flaveola*	*Eimeria* spp.	1/2
**ORDER PICIFORMES**		
*Pteroglossus castanotis*	*Eimeria* spp.	1/3
*Ramphastos toco*	*Eimeria* spp.	5/6
**ORDER PSITTACIFORMES**		
*Amazona aestiva*	-	-/2
*Ara ararauna*	Strongyloidea	1/5
	*Eimeria* spp.	1/5
*Psittacara leucophthalmus*	-	-/2
*Psittacula krameri*	-	-/1
**SAMPLE POOL ENCLOSURE C**	-	-/1
*Psittacara leucophthalmus*		
*Nandayus nenday*		
**SAMPLE POOL ENCLOSURE D**		
*Psittacara leucophthalmus*	-	-/1
*Diopsittaca nobilis*		
		
**Class Reptilia**		
**ORDER SQUAMATA**		
*Micrurus* sp.	-	-/1
**SAMPLE POOL ENCLOSURE E**		
*Chelonoidis carbonaria*	-	-/1
*Chelonoidis denticulata*		
		

Equal superscript letters identify mixed infections.

**Table 2:** Feces of free-living animals collected in the Paragem Municipal Natural Park, in the municipality of Dourados, Mato Grosso do Sul, and results of coproparasitological analyses carried out between 2022 and 2024.

**Hosts**	**Parasites identified**	**Positive/tested**
**Class Mammalia**		
**ORDER CARNIVORA**		
Unidentified Canid	-	-/1
**ORDER CHIROPTERA**		
*Artibeus lituratus*	Coccidia	1/6
**ORDER RODENTIA**		
*Dasyprocta azarae*	-	-/2
*Hydrochoerus hydrochaeris*	Strongyloidea^a^	2/3
	Coccidia^a^	1/3
**Class Reptilia**		
**ORDER SQUAMATA**		
*Salvator* sp*.*	-	-/1

Equal superscript letters identify mixed infections.

Oocysts of *Eimeria* spp. were identified in birds of the species *Ramphastos toco* Statius Müller, 1776, *Ara ararauna* Linnaeus, 1758, *Pteroglossus castanotis* Gould, 1834, *Sicalis flaveola* Linnaeus, 1766 and *Oryzoborus angolensis* Linnaeus, 1766. The highest prevalence of these parasites was observed in toucans (*R. toco*), with 83.33% of positive samples (5/6) for this species and 5.20% (5/96) of the total positive samples. Oocysts of *Cystoisospora* spp. were found in the carnivores *Cerdocyon thous* Linnaeus, 1766 and *Herpailurus yagouaroundi* Geoffroy, 1803. *Entamoeba* spp. were found in 8.33% (8/96) of the captive animals, mainly in some passerine birds and in a sample of *Tapirus terrestris* Linnaeus, 1758.

Eggs of the Strongyloidea superfamily were identified in 12.84% (14/109) of the analyzed samples; in 11.45% (11/96) of captive animals and 15.38% (2/13) of free-ranging animals samples. The positive animals were *Hydrochoerus hydrochaeris* Linnaeus, 1766, *Sapajus* spp*.* Linnaeus, 1758, *Didelphis albiventris* Lund, 1840, *Dicotyles tajacu* Linnaeus, 1758, *Artibeus lituratus* Olfers, 1818, *H. yagouaroundi*, *T. terrestris, C. thous*, and *A. ararauna.*

In the present study, eggs compatible with cestodes of the genus *Spirometra* were found in *Procyon cancrivorus* Cuvier, 1798 (1.04%, 1/96). In addition, *Dipylidium* spp. eggs were identified in the fecal samples collected from *H. yagouaroundi* in captivity. Regarding trematodes, *Paragonimus* spp. eggs were identified in fecal samples from two *Alouatta caraya* Humboldt, 1812 (2/96).

Regarding parasites with zoonotic potential, a significant number of helminths and protozoa were identified that can affect humans, such as the trematode *Paragonimus* spp.; the cestodes *Dipylidium* spp. and *Spirometra* spp.; the coccidia *Cystoisospora* spp. and *Entamoeba* spp.; and the nematodes *Toxocara* spp., the Ancylostomatidae family, *Trichuris* spp., Capillarinae, *A. suum*, and the Strongyloidea family.

## Discussion

The sampled wild animals belonged to the Cerrado, Atlantic Forest (Parque Natural Municipal do Paragem), and Pantanal biomes. The Cerrado and Pantanal are considered hotspots and rich and priority areas for global conservation (Myers et al., 2000). Factors such as intense crowding, space restrictions in captive animals, and confinement stress may have resulted in a higher incidence of parasitism in captive animals than in those living in the wild. Although the number of samples collected from free-ranging animals was small, the aforementioned factors should be considered. However, these findings are highly relevant considering that these animals do not receive any type of antiparasitic therapy, and infection secures the permanence and circulation of these parasites in the wild environment.

According to Dib et al. (2020), host identification can be done through taxonomic keys, molecular analysis and trichology. In this study, samples of free-living animals were collected from the environment and examined based on their appearance and morphology to identify hosts, although correct species determination was a limiting factor.

In the present study, 5.20% (5/96) of the samples were positive for *Eimeria* spp., with birds being the main host species. Like this study, Melo et al. (2022) found *Eimeria* oocysts in wild birds in captivity and in the wild at the Goiânia Zoo. Wild birds harbor a wide variety of parasites, the most frequent being protozoa from the coccidia group of the genera *Eimeria* and *Isospora* spp. (Santos et al., 2011). The protozoan *Eimeria* spp. are commonly found in the environment and can cause gastrointestinal disorders. Young animals are more prone to infection by this parasite (Peeters et al., 1984). Several factors contribute to the severity of the pathogenesis, such as the number of oocysts ingested, age of the host, presence and severity of other diseases, and nutritional status of the animal (Menezes, 2017). In high-density captive bird populations parasitic diseases are very common health problems (Barnes, 1986). Therefore, adequate management of enclosures and appropriate feeding and care with respect to overcrowding are fundamental for the control of eimeriosis in birds (Menezes, 2017). The high prevalence of *Eimeria* spp. in toco toucans in the investigation can be explained by the high population density in CRAS enclosures, which could have facilitated the spread of the protozoan.

*Cystoisospora* spp. oocysts were found in the carnivores *C. thous* and *H. yagouaroundi* which were under human care at CRAS. In another Wild Animal Screening Center (CETAS), located at the Zoo in Sapucaia do Sul, Rio Grande do Sul, Boll et al. (2017) found *Isospora* spp. in Passeriformes birds and Psittaciformes birds. Moreira et al. (2023) found oocysts of *Cystoisospora* spp. in feces of adult carnivorous mammals in two conservation institutions in the State of Goiás. Isosporiasis is a parasitic disease that affects several animal species and its pathogenicity depends on the animal's immune status (Peixoto et al., 2019). In the present study, no animal was showing symptoms of infection.

*Entamoeba* spp. were found in 8.33% (8/96) of the captive animals, mainly in some passerine birds and *T. terrestris*. These findings are similar to those of Prazeres et al. (2024) who demonstrated the presence of *Entamoeba* spp. cysts parasitizing captive exotic and wild birds in the Northeast region of Brazil. Species of the genus Entamoeba are morphologically similar and have adapted to live in the gastrointestinal tract of mammals and humans (Levine, 1985). Some enteric protozoa are known to have zoonotic potential, and humans are susceptible to infections by parasites that colonize the gastrointestinal tract (Thompson & Smith, 2011), such as the pathogenic amoeba *E. histolytica*, which is associated with intestinal and extraintestinal infections (CDC, 2019a). It is not yet known whether these species can cause infections in animals, but adequate sanitary control of the enclosures under the responsibility of the veterinary caretakers who work on site is very important.

Nematodes of the Strongyloidea family had the highest prevalence in this study; they were identified in 12.84% (14/109) of the analyzed samples. Sprenger et al. (2017) found that of the 110 fecal samples of birds and mammals from the State of Paraná, 65.5% (72/110) contained Strongyloidea eggs. In a study conducted at a zoo in Paraná, Snak et al. (2017) found that of 48 fecal samples of the genus *Cebus* Erxleben, 1777 (capuchin monkey), 37.5% (18/48) contained Strongyloidea eggs. In the current study, it was possible to classify these eggs only up to the family as described by Zajac & Conboy (2011) and Tavares et al. (2017). Barbosa et al. (2020) detected evolutionary forms of helminths in fecal material from non-human primates in Rio de Janeiro, compatible with the superfamily Ancylostomatoidea or superfamily Trichostrongyloidea/Strongyloidea. The difficulty in identifying parasites at the species level occurs due to the similarity between some eggs and oocysts, which can lead to the grouping of different parasites into a single species.

In the present study, eggs of the Ancylostomatidae family (hookworms) were identified in mammals as *A. caraya, Puma concolor* Linnaeus, 1771, *H. yagouaroundi, Leopardus pardalis* Linnaeus, 1758, *Chrysocyon brachyurus* Illiger, 1815, *C. thous* and *Lycalopex vetulus* Lund, 1982 including the subfamilies Ancylostomatinae; their genera *Ancylostoma* and *Uncinaria* are known to cause hemorrhage in the gastrointestinal tract of carnivores (Foster et al., 2006). Moreira et al. (2023) reported the presence of Ancylostomatidae and *Toxocara* spp. in *L. pardalis* in the State of Santa Catarina, in 21 *Leopardus wiendii* Schinz, 1821 and 14 *Leopardus tigrinus* Schreber, 1775. Kusma et al. (2015) also found Ancylostomatidae in 61.9% (13/21) of *L. wiendii* and 28.5% (4/14) of *L. tigrinus*, reporting a high prevalence. Agents that cause hemorrhage, which occurs recurrently in animals, especially captive animals, may pose a risk to endangered animals and species conservation processes.

*Trichuris* spp. eggs were detected in *P. cancrivorus* and *C. thous*. Trichurids have been reported in several regions of the country, as observed by Müller et al. (2005), who collected fecal samples from felines at the Pomerode Municipal Zoo, Santa Catarina, and the Brusque Zoobotanical Ecological Park Foundation and found the presence of *Trichuris* spp. in 30.7% and 23.1% of the animals, respectively. Braga et al. (2010) identified *Trichuris vulpis* in maned wolves (*C. brachyurus*) in the Emas National Park in the state of Goiás. Dib et al. (2020) conducted an epidemiological survey of gastrointestinal parasites in fecal samples of carnivores and artiodactyls in Itatiaia National Park (PNI) in the states of Minas Gerais and Rio de Janeiro, and found *Trichuris* spp., among other parasites.

In research carried out in the State of Goiás with captive animals, *Capillaria* spp., were identified in wild birds in captivity and in the wild at the Goiânia Zoo (Melo et al., 2022). In this study, Capillarinae eggs were found in captive *C. thous* at CRAS. Kusma et al. (2015) also found *Capillaria* spp. eggs in wild felines in Santa Catarina.

Some species of parasites are geohelminths, such as Strongyloidea, Ancylostomatidae, *Trichuris* spp., *Toxocara* spp. and *Ascaris suum,* normally found in soil. Sometimes the presence of these parasites in the feces of captive animals may be related to the collected environment and do not necessarily cause infection in the hosts (Barbosa et al., 2020). Snak et al. (2017) analyzed the frequency of parasites in wild mammals at the Cascavel Municipal Zoo, Paraná, also finding *Ancylostoma* spp., *Toxocara* spp. and *Trichuris* spp., as in this study. *Toxocara* spp. can be transmitted by paratenic hosts in wild environments (Carvalho & Rocha, 2011). In studies based on coproparasitological analyses of wild felines and other captive animals, *Toxocara* spp. were present in the samples studied (Silva et al., 2021; Uribe et al., 2021). The importance of these findings in the present study lies in the fact that toxocariasis is a zoonosis that occurs through the accidental ingestion of embryonated eggs from the soil, together with the feces of natural hosts, and can cause a variety of conditions from allergies to liver and lung damage (Campos et al., 2003). The presence of these geohelminths was identified in felines and canines assisted by CRAS. It is likely that the infections were acquired while the animals were still in the wild, suggesting the active circulation of these parasitic agents within their respective biomes.

Another highlight of parasites of zoonotic importance is the eggs of *A. suum* which were identified in two samples of *D. tajacu* (2/5). This is one of the most common parasitic diseases in pig husbandry worldwide (Roepstorff & Murrell, 1997). As reported by Castro et al. (2002), the presence of this parasite has already been detected in wild pigs in the Pantanal, indicating the risk of transmission to other pig species.

Species of the genus *Spirometra* were detected in a sample of *P. cancrivorus*. These are Diphyllobothriidae tapeworms with complex life cycles, with adult stages parasitizing the intestines of canines and felines, and can affect humans (Mueller, 1974). Dib et al. (2019) analyzed 213 fecal samples of free-living carnivores from Itatiaia National Park, Rio de Janeiro, and 52 (24.4%) tested positive for *Spirometra* spp. eggs. In humans, *Spirometra* spp. cause sparaganosis, an infection caused by the larvae of the parasite, which migrate to the muscles, subcutaneous tissue, and brain, forming masses that can cause neurological signs. Infection can occur through ingestion of *Spirometra* spp. eggs by copepods or contaminated intermediate hosts (Yang et al., 2022). The positive sample in this study was from an animal of the species *P. cancrivorus* that had coinfections and was recently rescued from Bonito, MS, a region with many rivers and tourist attractions.

*Dipylidium* spp. eggs were identified in fecal samples collected from a captive *H. yagouaroundi*. Gravid proglottids are excreted intact in the feces or may actively migrate from the perianal region of the host (CDC, 2019b). In most cases, the material eliminated in the feces consists predominantly of proglottids and not directly of eggs. Therefore, diagnosis by means of microscopic coproparasitological techniques may underestimate the real prevalence of the parasite, and also, due to the similarity of the eggs with other tapeworms (Luzio et al., 2015). However, studies with parasites identified by necropsy (the most sensitive technique for prevalence studies) in the same biomes reveal a low occurrence of the parasite in both domestic and wild felines (Ramos et al., 2013; Moraes et al., 2024), while in domestic canids (primary hosts) it is significantly higher (Ramos et al., 2015). It is the most common tapeworm in dogs and can also affect felines and humans through infected fleas (Yasuda et al., 1968). It causes dipylidiosis in humans, with a higher incidence in children who are generally asymptomatic (Santos, 2017). As this is a zoonosis, sanitary precautions must be taken to avoid infection, albeit of low clinical severity. One of the methods of controlling dipylidiosis in animals is to control intermediate hosts, using ectoparasiticides if necessary (ESCCAP, 2019).

*Paragonimus* spp. were also found in this study, parasitizing two *A. caraya*. Its definitive natural hosts include a wide range of wild carnivorous and omnivorous mammals including monkeys (Doanh et al., 2016). According to the CDC (2024b), more than 30 species have been reported in humans and animals and are found in the Americas, Southeast Asia, and Africa. Paragonimiasis is a zoonotic disease that causes lesions in the lungs of the host (Blair et al., 2007). Kusma et al. (2015) found eggs of *Paragonimus* spp. in the fecal samples of wild felines in the Flona de Três Barras in Santa Catarina. They also reported the presence of eggs of *Spirometra* spp., eggs of Ancylostomatidae and *Trichuris* spp., similar to the present study. The animals positive for *Paragonimus* spp. were Black Howler Monkey from the municipalities of Dourados and Campo Grande, MS, located in different enclosures at the time of sample collection.

## Conclusions

The occurrence of parasitic infections was significant, notably for parasites with zoonotic potential, such as the trematodes *Paragonimus* spp., cestodes *Dipylidium* spp., *Spirometra* spp., *Entamoeba* spp., and some nematodes. The nematodes Strongyloidea and Ancylostomatidae were identified in greater numbers.

Captive animals exhibited a higher prevalence of parasites than free-living animals. This higher prevalence may be due to the larger number of animals sampled, stress factors, and high population density in the enclosures of captive an imals. The spontaneous sedimentation technique that detects heavier parasite eggs showed the highest prevalence of eggs and oocysts in this study. Based on these results, we emphasize the importance of similar studies to better understand the circulation of etiological agents that may pose a risk to animal and human health.
